# COVID-19 vaccination requirements, encouragement and hesitancy among non-health care, non-congregate workers in Chicago: results from the WEVax survey

**DOI:** 10.1186/s12889-023-15781-x

**Published:** 2023-05-25

**Authors:** Frances R. Lendacki, Linda S. Forst, Supriya D. Mehta, Janna L. Kerins

**Affiliations:** 1 Chicago Department of Public Health, 1340 S. Damen Ave. 4th Floor, Chicago, IL 60608 USA; 2 School of Public Health, University of Illinois Chicago, 1603 W. Taylor St., Chicago, IL 60612 USA

**Keywords:** COVID-19 vaccines, Vaccine hesitancy, Non-healthcare workers, Employee health

## Abstract

**Background:**

While frontline and essential workers were prioritized for COVID-19 vaccination in the United States, coverage rates and encouragement strategies among non-health care workers have not been well-described. The Chicago Department of Public Health surveyed non-health care businesses to fill these knowledge gaps and identify potential mechanisms for improving vaccine uptake.

**Methods:**

The Workplace Encouragement for COVID-19 Vaccination in Chicago survey (WEVax Chicago) was administered using REDCap from July 11 to September 12, 2022, to businesses previously contacted for COVID-19 surveillance and vaccine-related outreach. Stratified random sampling by industry was used to select businesses for phone follow-up; zip codes with low COVID-19 vaccine coverage were oversampled. Business and workforce characteristics including employee vaccination rates were reported. Frequencies of requirement, verification, and eight other strategies to encourage employee vaccination were assessed, along with barriers to uptake. Fisher’s exact test compared business characteristics, and Kruskal–Wallis test compared numbers of encouragement strategies reported among businesses with high (> 75%) vs. lower or missing vaccination rates.

**Results:**

Forty-nine businesses completed the survey, with 86% having 500 or fewer employees and 35% in frontline essential industries. More than half (59%) reported high COVID-19 vaccination rates among full-time employees; most (75%) workplaces reporting lower coverage were manufacturing businesses with fewer than 100 employees. Verifying vaccination was more common than requiring vaccination (51% vs. 28%). The most frequently reported encouragement strategies aimed to improve convenience of vaccination (e.g., offering leave to be vaccinated (67%) or to recover from side effects (71%)), while most barriers to uptake were related to vaccine confidence (concerns of safety, side effects, and other skepticism). More high-coverage workplaces reported requiring (*p* = 0.03) or verifying vaccination (*p* = 0.07), though the mean and median numbers of strategies used were slightly greater among lower-coverage versus higher-coverage businesses.

**Conclusions:**

Many WEVax respondents reported high COVID-19 vaccine coverage among employees. Vaccine requirement, verification and addressing vaccine mistrust may have more potential to improve coverage among working-age Chicagoans than increasing convenience of vaccination. Vaccine promotion strategies among non-health care workers should target low-coverage businesses and assess motivators in addition to barriers among workers and businesses.

**Supplementary Information:**

The online version contains supplementary material available at 10.1186/s12889-023-15781-x.

## Background

As the COVID-19 pandemic has continued, vaccinations have proven critical to prevention of severe illness, hospitalization, and death. Recognizing the associations between workplace exposure and likelihood of contracting COVID-19, the Advisory Committee on Immunization Practices (ACIP) recommended that employees in critical infrastructure and highest-risk industries be prioritized for vaccination [1]. In March 2021, the U.S. Cybersecurity & Infrastructure Security Agency (CISA) cited vaccine hesitancy among these workers as detrimental to both the nationwide vaccine rollout and the continued functioning of the U.S. critical infrastructure [2].

The World Health Organization has defined vaccine hesitancy as “a delay or refusal to accept vaccines” despite their availability [3], with a “3 Cs” framework characterizing reasons as related to 1) a lack of confidence in vaccines, 2) inconvenience of being vaccinated, and 3) complacency about needing vaccination. While most studies of COVID-19 vaccine hesitancy by occupation have focused on health care workers, nationally-representative surveys conducted during early vaccine availability (March through June 2021) measured vaccine coverage and intent specifically among frontline essential and other non-health care workers [4–6]. Overall, these studies identified a lack of confidence (concerns of vaccine side effects, safety, and ineffectiveness) as an overarching reason for vaccine hesitancy; they cited that strategies to increase convenience of vaccination (providing on-site vaccination, or paid time off for vaccination and recovery) have potential to increase vaccination. At the time of this report, coverage rates remain sub-optimal among working-age Americans, despite broad availability of three FDA-approved COVID-19 vaccines, and updated evaluations of strategies to increase coverage are needed.

Chicago followed federal guidance for vaccine allocation, with frontline and other essential workers (Phases 1b and 1c) becoming eligible on January 25 and March 29, 2021 before general eligibility (Phase 2) on April 19, 2021 [7]. Mass vaccination sites and mobile vaccination programs were initially reserved for 1b/1c workers, before outreach became more targeted toward neighborhoods with low uptake despite high vulnerability to COVID-19. Questions probing COVID-19 vaccine hesitancy were incorporated into CDPH’s routine case investigation and contact tracing (CICT) for COVID-19 through the end of universal contact tracing in May of 2022 [8].

At the time of survey deployment, the rate of full vaccination in Chicago was 77% but as low as 48% in some zip codes [9]. Given the continuing need to inform vaccine promotion initiatives, CDPH conducted a study of workplace encouragement for COVID-19 vaccination (“WEVax Chicago”) from July—September of 2022. The survey aimed to describe frequency of vaccination requirements, encouragement strategies and persisting challenges to uptake among non-health care workplaces of varying sizes and industries throughout Chicago. This report summarizes the survey’s findings and implications for future research to improve vaccination coverage among non-health care workers.

## Methods

### Study design and population

The Workplace Encouragement for COVID-19 Vaccination in Chicago (WEVax Chicago) survey was a cross-sectional survey administered through REDCap [10] from July 11 through September 12, 2022, among businesses with at least one location in Chicago. The study excluded businesses classified as health care-related, government, or based in congregate settings (e.g., long-term care facilities, educational and childcare settings, shelters, and correctional facilities), given vaccination requirements and rollout strategies specific to these [11–14]. Survey respondents are thus described as non-health care, non-congregate workplaces (NHNCW) for the remainder of this report. NHNCW were categorized into 13 industry sectors for sampling, consistent with those used to summarize Chicago’s workplace COVID-19 surveillance data [15]. These included four early vaccine eligibility (“1b”) (Food Production & Processing, Manufacturing, Warehousing & Distribution, Grocery) and nine others (Bars & Restaurants, Construction, Retail, Hotel, Office Settings, Personal Care & Service, Janitorial, Transportation and Other) [7]. The sample included 537 businesses that had been previously contacted by CDPH for COVID-19 surveillance and vaccine-related outreach (e.g., follow-up on reported cases or potential workplace-related transmission among employees, or mobile vaccination efforts during early-phase vaccine rollout).

To improve response rate, two CDPH interviewers conducted active recruitment by calling just over one third (35%, 186/537) of businesses from the initial contact list, chosen through random sampling stratified by industry group for representativeness. Businesses located in zip codes with first-dose coverage rates below the citywide average were oversampled for phone outreach; these comprised 38% of all businesses called. For example, the citywide first-dose coverage rate on the date of survey deployment was approximately 77%, but as low as 55% in some zip codes according to CDPH’s vaccine data dashboards [9]. In all but two industry categories, at least two businesses selected for phone outreach were in low coverage regions. (For janitorial workplaces and hotels, CDPH had only one contact in a low-coverage zip code.) Within manufacturing, bars/restaurants, food production/processing, and transportation strata, at least half (≥ 50%) of workplaces called were in low-coverage zip codes. The survey (Additional file 1) was sent to five businesses during a pilot period the week before deployment for feedback on length, clarity, feasibility, and ease of answering questions. This study was determined to be exempt from review by the Institutional Review Board (IRB) at CDPH (Protocol #22–03).

### Workplace (business) and workforce (employee) characteristics

Industry of the responding business was collected as free-text, per NIOSH recommendations [16] (“How would you describe your primary type of business or industry?”). With closed-ended response categories, respondents were asked to indicate whether describing employees of multi-location businesses, or a single-location business (in which case zip code was also collected). Total full-time and part-time staff, proportion working off-site at time of survey, primary languages spoken, and availability of employer-sponsored health insurance were collected. (In this report, part-time or other temporary/contract staff are referred to collectively as “part-time staff”.) Workforce race and ethnicity data are not included, due to concerns around inaccuracies and missingness in reported data, potentially stemming from reluctance of businesses to disclose in relation to COVID.

### Estimation of COVID-19 vaccine coverage among employees

A definition of terms preceded the vaccination requirements section of the survey. “Primary series” of COVID-19 vaccination was defined as “the doses recommended for individuals to be considered "fully vaccinated" against COVID-19”. During the survey period, this included: 1) two doses of Pfizer-BioNTech given three to eight weeks apart, 2) two doses of Moderna given four to eight weeks apart, or 3) one dose of Johnson & Johnson’s Janssen vaccine. This survey was conducted before the availability of updated (“bivalent”) boosters, so did not distinguish between original and newer-formulation booster doses when assessing proportions of boosted employees. Businesses were asked to report employee vaccination and booster rates or the number of employees who had received their primary series and any booster doses. Among businesses that specified numbers instead of proportions of employees who were fully vaccinated and boosted, vaccination rates were calculated from reported total numbers of employees. Due to the small sample and degrees of missingness, rates were maintained as a categorical variable (lower vaccination coverage (≤ 75%), higher vaccination coverage (> 75%), missing).

### Vaccine requirement, encouragement strategies and barriers

Businesses were asked if they required employees to be 1) fully vaccinated and/or 2) boosted as eligible, and if vaccination status was verified. Verification method was collected using a free-text field. The survey also assessed any use of eight other strategies derived from CISA guidance for vaccine encouragement among essential workers (offering on-site vaccination, paid time off for vaccination or side effects, monetary or other incentive for vaccination, use of workplace signage or other communication tools to promote vaccination, training for staff to serve as vaccine ambassadors, and townhalls or information sessions to promote vaccination among workers) [2]. Free-text sections allowed respondents to describe other strategies and challenges to vaccine encouragement among employees.

### Analytic and statistical methods

All analyses were completed using SAS (version 9.4).

### Business characteristics

Vaccine eligibility was defined dichotomously by City-designated industry group, as frontline essential/early eligibility for vaccine (“1b”) [7] or other. While essential workers not included in 1b may have been vaccinated in the 1c phase preceding broad (“Phase 2”) eligibility in Chicago, most 1c and Phase 2 workers were vaccinated in the same period (April through June of 2021), compared to 1b workers (February and March of 2021). To aid comparison with findings from other jurisdictions, NIOSH’s Industry and Occupation Computerized Coding System (NIOCCS) was also used to categorize free-text industry descriptions into one of 27 major groupings per the North American Industry Classification System (NAICS) [17]. Business size was defined categorically from total number of staff. Zip codes were used to classify single-location businesses by city region, consistent with Healthy Chicago Equity Zones used by City departments for public health outreach and resource allocation [18].

### Vaccine requirement, encouragement strategies and barriers

Use of each encouragement strategy was dichotomized (any or never) for primary series and/or boosters, and among full-time and part-time employees separately. Mean (with standard deviation, SD) and median (with interquartile range, IQR) numbers of strategies reported per workplace were calculated. Bivariate analyses with Fisher’s exact test compared coverage rates (higher versus lower) among workplaces reporting and not reporting use of each strategy. The Kruskal–Wallis test compared distributions of the *number* of strategies reported by workplaces in each coverage group. The hypotheses for these comparisons were 1) that businesses reporting use of encouragement strategies would also report higher coverage, and 2) that high-coverage workplaces would report using a greater number of vaccine encouragement strategies. Businesses missing estimated vaccination rates were still retained in the sample, given overall aims of 1) describing any strategies that businesses have used to encourage vaccination, or 2) related barriers.

Thematic analyses of barriers to vaccine encouragement reported in free text responses utilized a deductive approach: descriptions of encouragement practices and barriers were classified using the “3 Cs” model of factors of vaccine hesitancy (complacency, confidence, and convenience) [3]. Potential factors related to confidence included safety (side effects), medical conditions or provider advice, other mistrust or anxiety (e.g., related to efficacy, government mistrust, philosophical or religious objections). Factors related to convenience included being too busy or lacking access (perceived cost, transportation, difficulty finding vaccine providers). Factors of complacency included workers not feeling the vaccine was necessary or perceiving that prior infection would be sufficiently protective against future SARS-CoV-2 infection.

## Results

### Characteristics of WEVax survey respondents and workforce

**Fig. 1:**
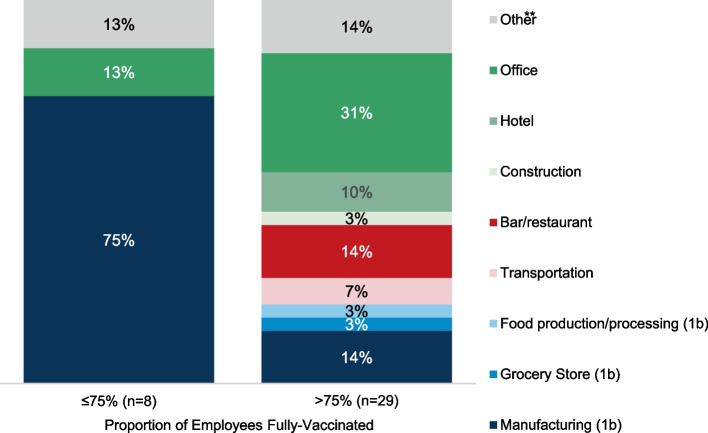
WEVax Chicago survey respondents with vaccination data (*n* = 37), workplace type by COVID-19 vaccination status* * “Vaccination status” refers to reported rates of full vaccination (initial series) among full-time employees only. Twelve respondents (not shown) did not report vaccination status but were retained in the final sample. ** “Other” workplaces (*n* =5): *n* =3 performing arts and *n* =1 veterinary in high coverage group, *n* =1 utilities company in low coverage group

From July 11 through September 12, 2022, survey response rates were 9% (49/537) among all e-mailed contacts, and 11% (21/186) among those called directly by CDPH; one additional respondent was recruited through social media. Among 50 total respondents, one out-of-jurisdiction business was excluded. Of the 49 workplaces included in the final sample, the most frequently reported titles of respondents were related to human resources (43%), followed by leadership positions (e.g., manager, chief executive officer, director, 39%). Few respondents (6%) specified job titles related to occupational health. The remainder (12%) reported financial or other job titles (accountant, payroll administrator, controller, general counsel, food safety manager), including one unknown. Thirty-seven respondents (76%) estimated rates of full vaccination (i.e., coverage rates) among full-time employees, and are described by workplace type and coverage level in Fig. 1. The distributions of characteristics of all 49 businesses in the final sample overall and by vaccine coverage are summarized in Table 1. Most respondents were in manufacturing and office settings (22%). Just over one third (35%) were in 1b industries. Classifications by major NAICS code are shown in supplemental material (Additional file 2, table). Most businesses were in North Central Chicago (43%) or West Chicago (29%); none were in the Near South Chicago region. Half had fewer than 100 employees (51%). Over half represented a single-location business (55%). Approximately three quarters (73%) said most of their staff were on-site. Spanish was the second most frequently reported primary language among employees (49%) after English (94%). Almost all (89%) reported sponsoring health insurance for full-time employees.

**Table 1:** Demographics of WEVax Chicago survey respondents (*n* = 49), overall and by estimated coverage rate among full-time employees

			% Full-time employees fully vaccinated					
	**All**		** ≤ 75%**		** > 75%**		**Missing**	
	**(*N* = 49)**		**(*n* = 8)**		**(*n* = 29)**		**(*n* = 12)**	
	n	(%)	n	(%)	n	(%)	n	(%)
**Eligibility phase**								
1b (early)	17	(34.6)	6	(75.0)	6	(20.6)	5	(41.6)
Other	32	(65.3)	2	(25)	23	(79.3)	7	(58.3)
**City region**								
North Central^a^	21	(42.9)	3	(37.5)	13	(44.7)	4	(33.3)
West	14	(28.6)	2	(25)	10	(34.4)	2	(16.6)
Northwest	7	(14.3)	2	(25)	4	(13.7)	1	(8.3)
Southwest	4	(8.2)	0	(–)	1	(3.4)	3	(25)
Far South	2	(4)	1	(12.5)	1	(3.4)	0	(–)
Multiple or unknown	1	(2)	0	(–)	0	(–)	2	(16.6)
**Workplace size**								
Fewer than 100	25	(51)	5	(62.5)	15	(51.7)	5	(41.6)
100–500	17	(34.6)	2	(25)	12	(41.3)	3	(25)
Over 500 employees	5	(10.2)	1	(12.5)	2	(6.8)	2	(16.6)
Unknown	2	(4)	0	(–)	0	(–)	2	(16.6)
**% of full-time employees teleworking**								
0	21	(42.9)	5	(62.5)	10	(34.5)	6	(50)
1–25%	15	(30.6)	1	(12.5)	10	(34.5)	4	(33.3)
26–100%	13	(26.5)	2	(25)	9	(31)	0	(–)
NA or do not know	2	(4)	0	(–)	0	(–)	2	(16.7)
**Primary languages spoken in workplace**^**b**^								
English	46	(93.8)	8	(100)	29	(100)	9	(75)
Spanish	24	(48.9)	3	(37.5)	13	(44.8)	8	(66.6)
Other	12	(24.5)	2	(25)	8	(27.6)	2	(16.7)
**Employer-sponsored health insurance**^**c**^								
For full-time employees	42	(85.7)	8	(90)	26	(89.6)	10	(83.3)
For both full-time and part-time employees	2	(4)	0	(–)	2	(6.8)	0	(–)
No	1	(2)	0	(–)	1	(3.4)	0	(–)
Unknown	2	(4)	1	(12.5)	1	(3.4)	2	(16.6)
**Location types**								
Single (only) location	27	(55.1)	5	(62.5)	16	(55.1)	6	(50)
Multiple locations, combined	13	(26.5)	1	(12.5)	6	(20.6)	6	(50)
One of multiple	9	(18.3)	2	(25)	7	(24.1)	0	(–)

^a^One responded on behalf of employees across multiple locations of a business with one location in North Central Chicago, and other locations outside Illinois

^b^Other languages reported as primary languages spoken among workers: Polish: *n* = 4/49 (8%), Chinese including Mandarin and Cantonese: *n* = 4/49 (8%), Arabic: *n* = 3/49 (6%), Tagalog: *n* = 1/49, (2%); workplaces reported multiple primary languages spoken among workers, columns sum to greater than 100%

^c^Workplaces reported multiple responses about employer-sponsored health insurance (i.e., yes for full-time employees, unknown for others); columns sum to greater than 100%

### Employee vaccination requirements and coverage

Distributions of COVID-19 vaccine coverage estimates are shown in Table 2. Most businesses (59%) reported high rates of full vaccination among full-time staff. The eight workplaces reporting that 75% or fewer full-time staff were fully vaccinated were geographically diverse (Table 1); most were manufacturing facilities (75%) and had fewer than 100 full-time employees (63%). Forty-one percent of respondents did not report estimated rates of booster vaccination. About three quarters (76%) of respondents indicated having any part-time staff, but due to high levels of missing data, subsequent sections of this report focus on full vaccination and encouragement among full-time employees only; data on part-time employees are described in supplemental content (Additional file 3, table).

**Table 2:** Estimated COVID-19 vaccination coverage among full-time and part-time workers among WEVax Chicago survey respondents (*n* = 49)

		Full-time (*n* = 49)^a^	Part-time or other (*n* = 37)^b^
		n (% of non-missing)	n (% of non-missing)
Primary series	0%	0 (-)	1 (4.2)
	1–25%	0 (-)	0 (-)
	26–50%	6 (16.2)	2 (8.3)
	51–75%	2 (5.4)	1 (4.2)
	76–99%	21 (56.8)	10 (41.7)
	100%	8 (21.6)	10 (41.7)
Any boosters	0%	1 (3.4)	1 (5.9)
	1–25%	3 (10.3)	0 (-)
	26–50%	8 (27.6)	5 (29.4)
	51–75%	7 (24.1)	1 (5.9)
	76–99%	5 (17.2)	3 (17.6)
	100%	5 (17.2)	7 (41.2)

^a^Number of businesses missing COVID-19 vaccination rate estimates for full-time employees: *n* = 12/49 for primary series (25%), *n* = 20/49 for boosters (41%)

^b^Number of businesses missing COVID-19 vaccination rate estimates for part-time or other employees: *n* = 13 for primary series (35%), *n* = 20 for boosters (54%), of *n* = 37 workplaces indicating having any part-time staff

### Vaccination requirement and encouragement strategies

**Fig. 2:**
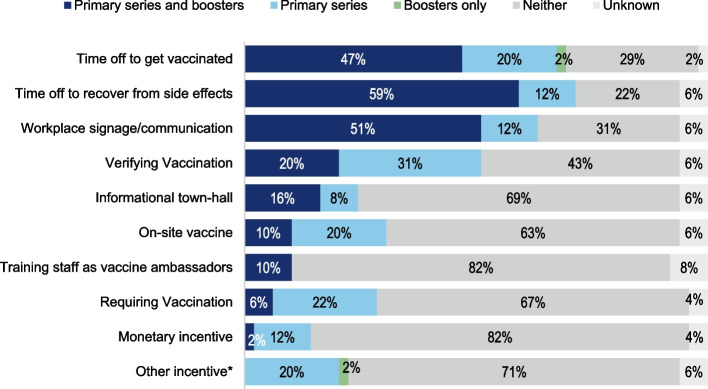
Strategies to encourage employee COVID-19 vaccination among WEVax Chicago survey respondents (*n* = 49) *Other incentives reported (10) included hiring preference for vaccinated candidates (among a high-coverage business), appointment assistance at city vaccination sites (among a low-coverage business), details were missing for one respondent

Frequencies of vaccine encouragement strategies are summarized in Fig. 2. Less than one third (28%) of businesses reported ever requiring employees to be vaccinated against COVID-19. Verifying vaccination was more common (51% or 25/49); 23/25 (92%) specified requesting vaccination card as a method of verification. All 14 businesses requiring vaccination and 11 others ever verified vaccination. Of those, five (20%) reported still doing so at the time of the survey. Among all respondents, providing time off to recover from side effects (71%), or to get vaccinated (69%) were the most frequently reported strategies, followed by use of promotional signage and communication (63%). Fifteen businesses (30%) reported offering vaccine on-site; 12 (24%) reported organizing informational townhalls, seven (14%) offered monetary incentives, five (10%) reported training staff as vaccine ambassadors. Eleven (22%) described other strategies aimed at convenience (sign-up or transportation, vaccine events with neighboring companies and at city-run sites). Non-monetary incentives included access to work-related social events and prioritization for job openings. Some respondents said employees got vaccinated to comply with requirements at client sites, or to protect coworkers.

Bivariate analyses of strategies reported among high versus lower-coverage workplaces are shown in Table 3. Thirteen of 14 (93%) businesses requiring full vaccination also reported high coverage, compared to 16 of 33 (49%) without this requirement (*p* = 0.03; one business requiring full vaccination did not provide any coverage estimates). Among businesses that reported verifying vaccination, almost all had high coverage rates (84%, 21/25), compared to 38% (8/21) among businesses that never verified vaccination (*p* = 0.07). Most businesses missing coverage data (8/12 missing rates of vaccination among full-time employees) reported not verifying vaccination. Lower-coverage workplaces reported a slightly higher median number of encouragement strategies compared to higher-coverage and those missing data (*p* = 0.12).

**Table 3:** Strategies to encourage employee COVID-19 vaccination among WEVax Chicago survey respondents, overall and by coverage rate (*n* = 49)

	*% Full-time staff fully vaccinated*			
	** ≤ 75% (*n* = 8)**	** > 75% (*n* = 29)**	**Missing** **(*n* = 12)**	*p* value*
	n, (%)	n, (%)	n, (%)	
**Require vaccination**				
Yes	0 (-)	13 (92.9)	1 (7.1)	0.03
No	8 (24.2)	16 (48.5)	9 (27.3)	
Unknown	0 (-)	0 (-)	2 (100)	
**Verify vaccination status**				
Yes	2 (8.0)	21 (84)	2 (8.0)	0.07
No	5 (23.8)	8 (38.1)	8 (38.1)	
Unknown	1 (33.3)	0 (-)	2 (66.7)	
**On-site vaccine**				
Yes	4 (26.7)	7 (46.7)	4 (26.7)	0.23
No	4 (12.9)	20 (64.5)	7 (22.6)	
**Paid time off to vaccinate**				
Yes	6 (18.2)	19 (57.6)	8 (24.2)	1.0
No	2 (13.3)	10 (66.7)	3 (20)	
**Paid time off to recover**				
Yes	8 (22.9)	21 (60)	6 (17.1)	0.31
No	0 (-)	7 (63.6)	4 (36.3)	
**Monetary**				
Yes	1 (14.3)	5 (71.4)	1 (14.3)	1.0
No	7 (17.5)	24 (60)	9 (22.5)	
**Workplace signage**				
Yes	6 (19.4)	19 (61.3)	6 (19.4)	1.0
No	2(13.3)	9 (60)	4 (26.7)	
**Vaccine ambassadors**				
Yes	1 (20)	3 (60)	1 (20)	1.0
No	7 (17.5)	25 (62.5)	8 (20)	
**Townhalls**				
Yes	4 (33.3)	7 (58.3)	1 (8.3)	0.20
No	4 (11.8)	22 (64.7)	8 (23.5)	
**Other incentivesa**				
Yes	0 (10.0)	7 (70.0)	2 (20.0)	0.31
No	8 (21.6)	21 (56.8)	8 (21.6)	
Unknown	0 (-)	1 (33.3)	2 (66.7)	
**Number strategies**				
Mean (SD)	3.75 (1.7)	3.0 (1.5)	2.4 (0.9)	0.12
Median (IQR)	4 (2–5)	3 (2–4)	2 (2–3)	

^*^p-values from Fisher’s exact test comparing workplaces with high and low vaccination rates, except in comparison of number of strategies reported (Kruskal–Wallis p-value for workplaces reporting high vs. low vs. missing coverage)

^a^Data were missing on any use of on-site vaccine, promotional signage, townhalls (*n* = 3/49, 6% each), time off to be vaccinated (*n* = 1/49, 2%) or recover (*n* = 3/49, 6%), monetary incentive (*n* = 2/49, 4%), vaccine ambassadors (*n* = 4/49, 8%) or other incentives (*n* = 3/49, 6%)

^b^Other incentives reported included (among highly-vaccinated business): conversations, access to work-sponsored social events, (missing coverage levels): hiring preference for vaccinated candidates; *n* = 1 was missing more information

### Vaccination barriers, challenges and hesitancy

From free text responses, multiple businesses reported that requiring vaccination was a challenge given already-existing difficulties with employee retention and unwillingness to end teleworking. Among descriptions of other barriers to encouragement of employee vaccination (Table 4), the primary theme was a lack of confidence in vaccines. One company cited complacency among employees who had already recovered from SARS-CoV-2 infection.

**Table 4:** Reasons for COVID-19 vaccine hesistancy among NHNCW, as reported by businesses responding to WEVax Chicago survey (*n* = 49)

**Theme**	**Subcategories**	Examples
Confidence	**Safety**	•Fear of side effects •Perceptions of mRNA vaccines as unsafe compared to older vaccines •Claims that family members died soon after receiving vaccine
	**Other mistrust, skepticism, or anxiety**	•Feeling that vaccination is too politicized •(government mistrust) •Hesitancy to work at a company that requires vaccination •Misinformation about life insurance policy cancellation •Conspiracies of vaccines containing implanted devices •Religious objections •Disbelief that COVID-19 is real •Skepticism of frequently changing CDC guidance
Complacency	**Already had COVID-19**	•Belief that natural immunity obviates need to vaccinate

## Discussion

The WEVax survey had three major findings regarding 1) vaccine requirements, 2) encouragement strategies, and 3) persisting barriers to workforce vaccination. Having a requirement for employee vaccination appeared to be associated with greater likelihood of achieving high vaccination coverage rates. Almost all respondents indicated use of multiple strategies for encouragement of vaccination, usually to increase the convenience of vaccination (offering time off to be vaccinated or recover, providing transportation, facilitating appointments). While themes of reported vaccine hesitancy centered around low vaccine confidence (personal concerns of vaccine safety, misinformation, and other skepticism among workers), initiatives to improve confidence and reduce complacency (vaccine ambassador training, informational town halls) were the least-frequently reported by WEVax survey respondents. Respondents did not indicate reasons for not employing these strategies, and barriers to their use have not been widely reported. In their summary of six virtual town halls encouraging COVID-19 vaccination among racial and ethnic minority groups in South Florida, Wagner et al. noted that these efforts were resource intensive and may have resulted in only small increases in likelihood to vaccinate among highly-vaccinated populations [19].

Though not collected concomitantly with the WEVax survey, individual-level CICT data collected by CDPH from June 2021—May 2022 (Additional file 4, figure) echo our findings. Vaccine confidence and misinformation about vaccine safety were identified as primary reasons for hesitancy, while inconvenience was reported far less frequently and decreasingly over time. This is important because it suggests that the encouragement strategies reported by workplaces may not directly address prevailing reasons for hesitancy as described by both workplaces and working-age Chicagoans. Furthermore, 41% of unvaccinated working-age Chicagoans refused to specify reasons for *not* vaccinating, suggesting that assessment of potential *motivators. *(e.g. – “what would it take for you to change your mind about being vaccinated for COVID-19”?) instead of barriers alone may generate more actionable data for increasing coverage rates among NHNCW. For example, the longitudinal HEROES RECOVER study conducted in 2020 found that increases in COVID-19 vaccine knowledge, safety and effectiveness were positively associated with intent to vaccinate among essential workers [20]. In their 2021 report, Nguyen et al. also described top motivators for COVID-19 vaccination by worker group [4]. Among non-health care frontline workers, the most frequently specified motivators were more data on vaccine effectiveness (29%) and safety (37%), workplace vaccination requirements (27%), and prevention of transmission to family and friends (31%) or in the community (21%), with similar findings among other non-health care workers. COVID-19 vaccines have received FDA approval since these studies have been conducted, and hospitalization and mortality rates have decreased substantially. Updated assessments could elucidate whether vaccine effectiveness, safety, and desire to protect others should still be considered key motivators for vaccination, or whether other messaging may be more effective at this phase of the pandemic.

The WEVax study was limited by low response rate (9%), though direct outreach by phone increased responsiveness. Response rate might have been higher if the survey had been conducted earlier in the pandemic when employers were more engaged with vaccination efforts. Low-coverage regions were underrepresented despite oversampling. Sampling was based on low rates of vaccine initiation instead of vaccine completion (i.e., first-dose versus full coverage rates), to target regions where residents may be in greatest need of vaccine encouragement. This contrasts with the primary metric described in this survey, full vaccination, which is more likely to be recorded or verified by businesses. In either case, metrics describing coverage among residents may not have been representative of workplaces in the same regions. It is possible that workplaces that are less promotive of workplace vaccination (whether by requirement or incentivization) or with poor coverage rates were less likely to participate. In addition, selection bias may have resulted in overestimates of high vaccine coverage and encouragement strategies used, since contact lists were comprised of businesses already willing to engage with CDPH for COVID-19 vaccination and prevention efforts. Survey data were subject to recall limitation, in that respondents may not have remembered (or been present for) encouragement strategies practiced by their workplaces previously. Of the 37 businesses estimating employee vaccination rates, 46% did not specify use of vaccination cards to verify vaccination status. Their estimation and verification methods are unknown, and misclassification may have occurred. Of these, 10 (59%) estimated high coverage rates and seven (41%) estimated low coverage rates. Differential misclassification may have occurred among businesses that reported never verifying vaccination status; these had greater missingness of coverage data (38% (8/21) compared to 8% (2/25) among those who checked vaccination status). Because so many businesses with part-time employees did not provide coverage estimates (65% (24/37) estimated full-vaccination rates, 46% (17/37) estimated rates of booster doses), we could not address knowledge gaps related to vaccination policies and promotion among these types of workers. Reasons for this missingness were unknown; it is possible that businesses had less administrative oversight of these workers compared to full-time staff. Finally, incomplete data on workforce demographics prevented identification of demographic groups that would benefit from targeted workplace-based messaging and outreach to improve vaccination.

Though limited, results from the WEVax survey may be useful in informing larger studies, and among small business outreach organizations like BACP in Chicago: 85% of respondents were small businesses (500 or fewer employees). The finding that higher-coverage workplaces reported a lower median number of encouragement strategies suggests that specific types of strategies, such as vaccine requirement and verification, are more strongly associated with increased coverage than others; use of more strategies is not necessarily associated with higher coverage. However, the temporality of encouragement strategies and vaccine coverage cannot be established given the cross-sectional nature of the survey. Larger prospective studies including a greater proportion of under-vaccinated workplaces could provide insights into approaches that have been differentially successful in highly vaccinated settings. These and future efforts to describe vaccine hesitancy at the individual level should include standardized collection of industry and occupation information, to facilitate classification (e.g., using NIOCCS) and stratification to describe NHNCW specifically.

Frequent allotment of time off to be vaccinated or recover from vaccination is unsurprising, given Chicago’s Vaccine Anti-Retaliation Ordinance passed in March 2021 [21]: businesses must allow workers to use allotted sick time or paid time off to be vaccinated against COVID-19, and those requiring employee vaccination must provide paid time off for employees to be vaccinated. Infrequent vaccination requirement is consistent with updated data from the KFF Vaccine Monitor Survey (April 13–26, 2022), indicating that only 40% of respondents said their workplaces required vaccines after withdrawal of federal vaccine mandates in January 2022. The WEVax survey did not ask respondents to specify the amount of any monetary incentives offered. A 2021 study conducted at a large manufacturing company in Minnesota found that from August through September 2021, a substantial ($1000) financial incentive increased employee vaccination rates from 76 to 86%, citing the limitations of no control group, and FDA-approval of the Pfizer vaccine during the study period [22]. Such incentives are not likely to be sustainable among smaller workplaces, or to outweigh all skepticism about safety and intention of vaccination efforts to combat COVID-19. A lack of other available data evaluating encouragement among non-health care workplaces in the U.S. highlights the need for future studies in these areas.

While the Center for Medicare and Medicaid Services vaccine mandate for health care workers was upheld by court challenges, the U.S. Supreme Court overturned the Occupational Safety and Health Administration (OSHA)’s emergency temporary standard for health care which required not only vaccines, but also masking and regular testing; the Supreme Court also disallowed these requirements for non-health care workers. This study demonstrates the practices of, likely, the most compliant companies in Chicago, and is instructive of what to expect without a national mandate for employers. While employers may not maintain oversight of workforce vaccination, public health agencies can help educate them on the connection between reducing risk in the workplace and surrounding communities.

## Conclusions

Most workplaces that responded to the WEVax survey reported high vaccination coverage against COVID-19, use of workplace communication to promote vaccination, and multiple strategies to increase convenience of getting vaccinated. Persisting vaccine mistrust and safety concerns were found to be greater barriers to vaccination than convenience among working-age Chicagoans in both workplace and individual-level analyses. Requirement and verification of vaccination were more common among high-coverage workplaces. Future studies to identify mechanisms for increasing vaccination among workers should include, at minimum, increased recruitment of low-coverage workplaces and assessment of potential motivators (in addition to barriers) among unvaccinated workers.

## Supplementary Information

**Additional file 1. **Draft of WEVax Chicago survey.

**Additional file 2. **WEVax Chicago survey respondents (*n *=49), by major industry sector.

**Additional file 3. **Strategies to encourage employee COVID-19 vaccination among WEVax Chicago survey respondents (*n*=49). 

**Additional file 4. **Primary reasons for not initiating COVID-19 vaccination.

## Data Availability

Datasets generated during the current study are not publicly available as these contain identifying information for responding businesses. De-identified data are available from the corresponding author on reasonable request.

## Abbreviations

BACP: Business Affairs and Consumer Protection
CDPH: Chicago Department of Public Health
CICT: Case Investigation and Contact Tracing
CISA: Cybersecurity and Infrastructure Security Agency
FDA: Food and Drug Administration
IQR: Interquartile Range
IRB: Institutional Review Board
KFF: Kaiser Family Foundation
NAICS: North American Industry Classification System
NHNCW: Non-health care, Non-Congregate Workplaces
NIOCCS: NIOSH Industry and Occupation Computerized Coding System
NIOSH: National Institute for Occupational Safety and Health
OSHA: Occupational Safety and Health Administration
SD: Standard Deviation
WEVax Chicago: Workplace Encouragement for COVID-19 Vaccination in Chicago
