# Supplementary material for: COVID-19 vaccination requirements, encouragement and hesitancy among non-health care, non-congregate workers in Chicago: results from the WEVax survey
Source: BMC Public Health. 2023 May 25;23:951. doi: 10.1186/s12889-023-15781-x (PMC10209568; doi:10.1186/s12889-023-15781-x)
Supplement: Supplementary file 1 — Additional file 1. Draft of WEVax Chicago survey. [file 12889_2023_15781_MOESM1_ESM.pdf]

Workplace Encouragement for COVID-19 Vaccination in Chicago
(The WEVax Chicago Study)Â

CDPH is studying how businesses encourage or require COVID-19 vaccination among employees, and what we can do to support these efforts.

Please help us by completing this brief survey.
Results will be shared in Fall of 2022.

All responses will be summarized in aggregate.
No businesses will be named when we present our findings
For questions/comments, contact workplaceCOVID@cityofchicago.org.

Please have this information at hand to help you complete the survey, if possible:

- workforce vaccination status
(number or estimated percentage of employees vaccinated)

- workforce demographics (optional)
(primary languages spoken, estimated breakdown by race and ethnicity groups)

Submission Date ________________________________

Business (Workplace) Characteristics

Business Name ______ Respondent Name ______
Business Address
(OK to leave blank, if describing employees at multiple locations in Chicago) ______ Respondent Title
(job title) ______
Business City ______ Respondent Phone Number ______
Business Zipcode ______ Respondent E-mail Address ______

How would you describe your primary type of business or industry? ________________________________
(For example: construction, restaurant, food processing, grocery store)

Please choose from one of the following options before describing the number and vaccination status of your employees:

- ◯ Our business has multiple locations. My answers describe employees who report to one of these locations
- ◯ Our business has multiple locations. My answers describe employees who report to multiple locations, combined
- ◯ Our business has one location

Approximately how many full-time employees are in this workplace? ________________________________

Approximately how many part-time, contract, and/or temporary employees are in this workplace? ________________________________

Approximately what percent of your full-time employees are off-site or teleworking?	◯ 0%<br>◯ 1-25%<br>◯ 26-50%<br>◯ 51-75%<br>◯ 76-99%<br>◯ 100% (all employees)<br>◯ Our workplace doesn't have full-time employees
Approximately what percent of your part-time/contract employees are off-site or teleworking?	◯ 0%<br>◯ 1-25%<br>◯ 26-50%<br>◯ 51-75%<br>◯ 76-99%<br>◯ 100% (all employees)<br>◯ Our workplace doesn't have these types of employees
Does your workplace require employees to be fully-vaccinated against COVID-19 and/or boosted, if eligible?	◯ Yes, fully vaccinated (primary series)<br>◯ Yes, fully vaccinated and boosted as eligible<br>◯ No vaccination requirement<br>◯ Unsure
Does your workplace check employees' COVID-19 vaccination status?	◯ Yes, for primary series (full vaccination)<br>◯ Yes, for primary series and boosters<br>◯ Our workplace does not check employees' vaccination status<br>◯ Unsure
If yes, how do you verify COVID-19 vaccination status?	__________________________________
Approximately when was this policy last implemented?	__________________________________<br>((Estimate using month/year, or provide other details if needed))
How would you like to report vaccination status of your full-time workforce?	◯ I can estimate the number of full-time employees who have been vaccinated<br>◯ I prefer to estimate a percentage of full-time employees who have been vaccinated<br>◯ Our workplace doesn't have full-time employees<br>◯ I cannot estimate the number or percentage of these employees who are vaccinated (I don't know)
How many of your full-time employees have completed their primary vaccination series?	__________________________________
How many of your full-time employees have received a booster shot?	__________________________________

	0%	1-25%	26-50%	51-75%	76-99%	100% (all employees)	Don't know

Approximately what percentage of your full-time workforce has received their primary series (is fully-vaccinated)?	◯	◯	◯	◯	◯	◯	◯
Approximately what percentage of your full-time workforce has received any booster shots?	◯	◯	◯	◯	◯	◯	◯

How would you like to report vaccination status of your part-time or other employees who are not full-time?

- ◯ I can estimate the number of part-time/other employees who have been vaccinated
- ◯ I prefer to estimate a percentage of part-time/other employees who have been vaccinated
- ◯ Our workplace doesn't have these other types of employees
- ◯ I cannot estimate the number or percentage of these employees who are vaccinated (I don't know)

How many of your part-time/contract/temporary employees have received a booster shot?

____

(leave blank, if you do not have these types of staff.)

How many of your part-time/contract/temporary employees have completed their primary vaccination series?

____

(leave blank, if you do not have these types of staff.)

	0%	1-25%	26-50%	51-75%	76-99%	100% (all employees)	Don't know
Approximately what percentage of your part-time/other workforce has received their primary series (is fully-vaccinated)	◯	◯	◯	◯	◯	◯	◯
Approximately what percentage of your part-time/other workforce workforce has received any booster shots	◯	◯	◯	◯	◯	◯	◯

Please indicate whether your workplace has ever used each strategy to encourage COVID-19 vaccination among employees.

In the dropdown options, "primary series" refers to the doses recommended for individuals to be considered "fully-vaccinated" against COVID-19. As of July 2022, this includes:

2 doses of Pfizer-BioNTech given 3â€“8 weeks apart
2 doses of Moderna given 4â€“8 weeks apart
1 dose of Johnson & Johnsonâ€™s Janssen

For Full-Time Staff
Choose 'Not Applicable'
if you do not have full-time staff
For Part-Time Staff
Choose 'Not Applicable'
if you do not have part-time or other types of staff

Offering vaccine on-site (in the workplace) ______ ______
Paid time off for receiving COVID-19 vaccinations ______ ______
Paid time off for recovering from side effects after receipt of COVID-19 vaccinations ______ ______
Monetary incentive (bonus) for receipt of COVID-19 vaccination ______ ______
Other incentive for receipt of COVID-19 vaccination ______ ______
Social media, internal communications, posters or signage around the workplace to encourage COVID-19 vaccination ______ ______
Training for interested staff to become COVID-19 vaccination ambassadors, share personal stories about the vaccine with fellow co-workers, address concerns ______ ______
Townhalls or other opportunities for leadership, respected local medical experts, and staff to share their COVID-19 vaccine experience, facts about the vaccine ______ ______

If your workplace has used other strategies to encourage COVID-19 vaccination among workers, please describe them here: ________________________________

We want to hear about challenges workplaces have experienced regarding requiring, enforcing, or encouraging vaccination among employees. Please share any details about these topics here: ________________________________

CDPH may follow-up with businesses about support for COVID-19 vaccination among workers.
Would your business like to be contacted about these topics?

- ☐ Yes, we would be willing to participate in a short, individual follow-up call with CDPH about these topics
- ☐ Yes, we would be willing to participate in a focus group with other businesses about these topics
- ☐ We would be interested in attending a webinar or seminar about these topics
- ☐ We do not wish to participate in a follow-up discussion about these topics

(Please check all that apply.)

Would you like to receive a copy of the survey findings when available?
(the e-mail address you entered at the beginning of this survey will be used)

- ☐ Yes, please send us a summary of the results from this survey when they are available.

Employee (Workforce) Demographics

Approximately what percent of your workforce identifies as each of these race-ethnicity groups? (Optional)

	0%	1-25%	26-50%	51-75%	76-99%	100% (all employees)	Don't know

Latinx (including those who identify as Latinx AND another race/ethnicity)	◯	◯	◯	◯	◯	◯	◯
Black, non-Latinx	◯	◯	◯	◯	◯	◯	◯
White, non-Latinx	◯	◯	◯	◯	◯	◯	◯
Asian, non-Latinx	◯	◯	◯	◯	◯	◯	◯
Other, non-Latinx (includes more than one race, but not Latinx at all)	◯	◯	◯	◯	◯	◯	◯

Primary language(s) spoken by employees

- ☐ English
- ☐ Spanish
- ☐ Polish
- ☐ Arabic
- ☐ Chinese (including Mandarin and Cantonese)
- ☐ Tagalog
- ☐ Other

(Check all that apply.)

If other, please specify

____

Does your workplace offer health insurance for employees?

- ◯ Yes, for full-time employees
- ◯ Yes, for part-time, temporary and/or contract employees
- ◯ Yes, for both full-time and part-time employees
- ◯ Yes, with another arrangement
- ◯ No
- ◯ Unknown

Please use this space for other details you wish to share about health insurance offered to employees of your workplace

____
