# Supplementary material for: COVID-19 vaccination requirements, encouragement and hesitancy among non-health care, non-congregate workers in Chicago: results from the WEVax survey
Source: BMC Public Health. 2023 May 25;23:951. doi: 10.1186/s12889-023-15781-x (PMC10209568; doi:10.1186/s12889-023-15781-x)
Supplement: Supplementary file 2 — Additional file 2. WEVax Chicago survey respondents (n =49), by major industry sector. [file 12889_2023_15781_MOESM2_ESM.docx]

ADDITIONAL FILE 2. WEVAX CHICAGO SURVEY RESPONDENTS (n=49),
BY MAJOR INDUSTRY SECTOR (NAICS) ^a^

When workplaces were classified by major NAICS code, manufacturing (both food and non-food) and accommodation and food services (hospitality) were most frequently represented (n=14, 29% and n=11, 23% of respondents, respectively).

| Industry Group | n (%) |
| --- | --- |
| Manufacturing | 14 (28.6) |
| Accommodation and Food Services | 11 (22.5) |
| Professional, Scientific and Technical Services | 6 (12.2) |
| Wholesale Trade | 3 (6.1) |
| Arts, Entertainment, Recreation | 2 (4.1) |
| Finance and Insurance | 2 (4.1) |
| Retail Trade | 2 (4.1) |
| Utilities | 2 (4.1) |
| Construction | 1 (2.0) |
| Educational Services | 1 (2.0) |
| Health Care and Social Assistance | 1 (2.0) |
| Information | 1 (2.0) |
| Management of Companies and Enterprises | 1 (2.0) |
| Real Estate and Rental and Leasing | 1 (2.0) |
| Transportation and Warehousing | 1 (2.0) |

*^a^NAICS: North American Industry Classification System designations. Free-text descriptions from WEVax survey respondents were classified using NIOCCS (the Industry and Occupation Computerized Coding System) designed by National Institute for Occupational Safety and Health, 2022 version.*
