# Supplementary material for: COVID-19 vaccination requirements, encouragement and hesitancy among non-health care, non-congregate workers in Chicago: results from the WEVax survey
Source: BMC Public Health. 2023 May 25;23:951. doi: 10.1186/s12889-023-15781-x (PMC10209568; doi:10.1186/s12889-023-15781-x)
Supplement: Supplementary file 3 — Additional file 3. Strategies to encourage employee COVID-19 vaccination among WEVax Chicago survey respondents (n=49). [file 12889_2023_15781_MOESM3_ESM.docx]

ADDITIONAL FILE 3. STRATEGIES TO ENCOURAGE EMPLOYEE COVID-19 VACCINATION AMONG WEVAX CHICAGO SURVEY RESPONDENTS (n=49)

(*continued on next page)*

|  |  | **Full-time** | | **Part-time** | |
| --- | --- | --- | --- | --- | --- |
|  |  | n (%) | % | n (%) |  |
| **Requiring Vaccination** | Primary series | 11 (22.5) | |  |  |
| (Assessed once for all types of workers) | Primary and boosters | 3 (6.1) |  |  |  |
|  | No | 33 (67.4) | |  |  |
|  | Unknown/not sure | 2 (4.1) |  |  |  |
| **Verifying Vaccination** | Primary series | 20 (0.4) |  | - |  |
| (Assessed once for all types of workers) | Primary and boosters | 15 (0.3) |  | - |  |
|  | No | 10 (0.2) |  | - |  |
|  | Unknown/not sure | 3 (0.1) |  | - |  |
| **On-site vaccine** | Primary series | 10 (20.4) | | 6 (16.2) |  |
|  | Primary and boosters | 5 (10.2) |  | 4 (10.8) |  |
|  | Boosters | 0 (0) |  | 0 (-) |  |
|  | Neither | 31 (63.3) | | 19 (51.4) |  |
|  | Not applicable | 3 (6.1) |  | 7 (18.9) |  |
|  | Unknown/not sure | - () |  | 1 (2.7) |  |
| **Time off to get vaccinated** | Primary series | 10 (20.4) | | 9 (24.3) |  |
|  | Primary and boosters* | 23 (46.9) | | 10 (27) |  |
|  | Boosters | 1 (2) |  | 1 (2.7) |  |
|  | Neither | 14 (28.6) | | 10 (27) |  |
|  | Not applicable | 1 (2) |  | 6 (16.2) |  |
|  | Unknown/not sure | 0 (-) |  | 1 (2.7) |  |
| **Time off to recover from side effects** | Primary series | 6 (12.2) |  | 4 (10.8) |  |
|  | Primary and boosters | 29 (59.2) | | 15 (40.5) |  |
|  | Boosters | 0 (0) |  | 0 (-) |  |
|  | Neither | 11 (22.4) | | 11 (29.7) |  |
|  | Not applicable | 1 (2) |  | 5 (13.5) |  |
|  | Unknown/not sure | 2 (4.1) |  | 2 (5.4) |  |

ADDITIONAL FILE 3. REASONS FOR COVID-19 VACCINE HESISTANCY AMONG NHNCW, AS REPORTED BY BUSINESSES RESPONDING TO WEVAX CHICAGO SURVEY (n=49) *(continued)*

|  |  | **Full-time** | | **Part-time** | |
| --- | --- | --- | --- | --- | --- |
|  |  | n (%) | % | n (%) |  |
| **Monetary incentive** | Primary series | 6 (12.2) |  | 3 (8.1) |  |
|  | Primary and boosters | 1 (2) |  | 0 (-) |  |
|  | Boosters | 0 (-) |  | 0 (-) |  |
|  | Neither | 40 (81.6) | | 29 (78.4) |  |
|  | Not applicable | 0 (0) |  | 4 (10.8) |  |
|  | Unknown/not sure | 2 (4.1) |  | 1 (2.7) |  |
| **Other incentive** | Primary series | 3 (6.1) |  | 1 (2.7) |  |
|  | Primary and boosters | 0 (-) |  | 2 (5.4) |  |
|  | Boosters | 1 (2) |  | 0 (-) |  |
|  | Neither | 42 (85.7) | | 28 (75.7) |  |
|  | Not applicable | 1 (2) |  | 5 (13.5) |  |
|  | Unknown/not sure | 2 (4.1) |  | 1 (2.7) |  |
| **Signage/communication in workplace** | Primary series | 6 (12.2) |  | 3 (8.1) |  |
|  | Primary and boosters | 25 (51.9) | | 16 (43.2) |  |
|  | Boosters | 0 (-) |  | 0 (-) |  |
|  | Neither | 15 (30.6) | | 13 (35.1) |  |
|  | Not applicable | 1 (2) |  | 4 (10.8) |  |
|  | Unknown/not sure | 2 (4.1) |  | 1 (2.7) |  |
| **Training staff to be vaccine ambassadors** | Primary series | 0 (-) |  | 0 (-) |  |
|  | Primary and boosters | 5 (10.2) |  | 2 (5.4) |  |
|  | Boosters | 0 (-) |  | 0 (-) |  |
|  | Neither | 40 (81.6) | | 29 (78.4) |  |
|  | Not applicable | 2 (4.1) |  | 5 (13.5) |  |
|  | Unknown/not sure | 2 (4.1) |  | 1 (2.7) |  |
| **Organizing an informational town-hall** | Primary series | 4 (8.2) |  | 3 (8.1) |  |
|  | Primary and boosters | 8 (16.3) |  | 5 (13.5) |  |
|  | Boosters | 0 (-) |  | 0 (-) |  |
|  | Neither | 34 (69.4) | | 24 (64.9) |  |
|  | Not applicable | 1 (2) |  | 4 (10.8) |  |
|  | Unknown/not sure | 2 (4.1) |  | 1 (2.7) |  |
