# Supplementary material for: COVID-19 vaccination requirements, encouragement and hesitancy among non-health care, non-congregate workers in Chicago: results from the WEVax survey
Source: BMC Public Health. 2023 May 25;23:951. doi: 10.1186/s12889-023-15781-x (PMC10209568; doi:10.1186/s12889-023-15781-x)
Supplement: Supplementary file 4 — Additional file 4. Primary reasons for not initiating COVID-19 vaccination. [file 12889_2023_15781_MOESM4_ESM.pdf]

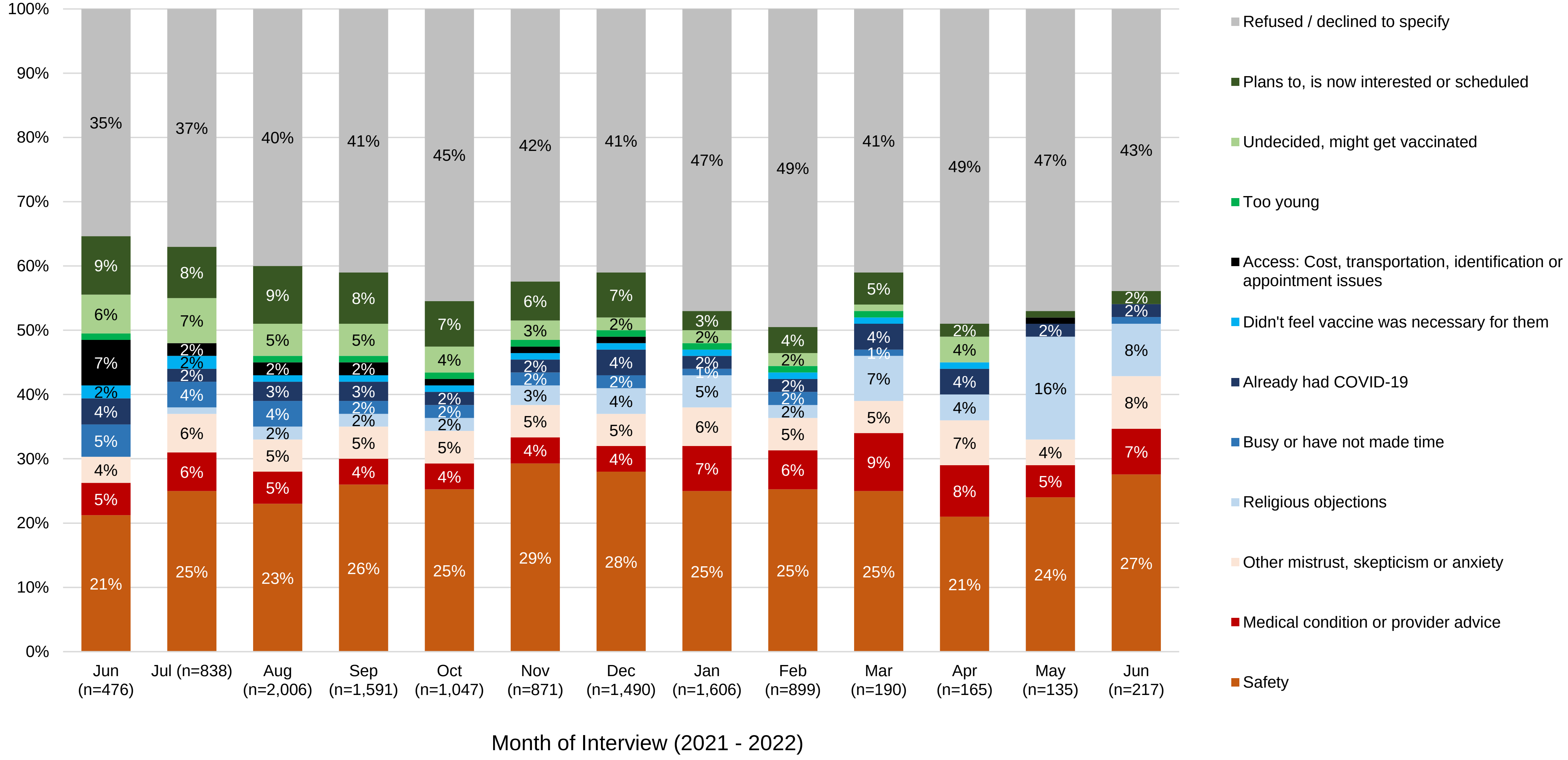

**Frequencies <2% not labeled*

Supplemental Figure 1. Primary Reasons for Not Initiating COVID-19 Vaccination among Unvaccinated Working-Age Chicagoans Interviewed June 1, 2021 – May 31, 2022 (n=9,925)*
